# Impaired 8-Hydroxyguanine Repair Activity of MUTYH Variant p.Arg109Trp Found in a Japanese Patient with Early-Onset Colorectal Cancer

**DOI:** 10.1155/2014/617351

**Published:** 2014-03-23

**Authors:** Kazuya Shinmura, Masanori Goto, Hong Tao, Hisami Kato, Rie Suzuki, Satoki Nakamura, Tomonari Matsuda, Guang Yin, Makiko Morita, Suminori Kono, Haruhiko Sugimura

**Affiliations:** ^1^Department of Tumor Pathology, Hamamatsu University School of Medicine, 1-20-1 Handayama, Higashi Ward, Hamamatsu, Shizuoka 431-3192, Japan; ^2^Division of Cancer Development System, National Cancer Center Research Institute, Tokyo 104-0045, Japan; ^3^Research Center for Environmental Quality Management, Kyoto University, Shiga 520-0811, Japan; ^4^Department of Preventive Medicine, Graduate School of Medical Sciences, Kyushu University, Fukuoka 812-8582, Japan

## Abstract

*Purpose.* The biallelic inactivation of the 8-hydroxyguanine repair gene *MUTYH* leads to MUTYH-associated polyposis (MAP), which is characterized by colorectal multiple polyps and carcinoma(s). However, only limited information regarding MAP in the Japanese population is presently available. Since early-onset colorectal cancer (CRC) is a characteristic of MAP and might be caused by the inactivation of another 8-hydroxyguanine repair gene, *OGG1*, we investigated whether germline *MUTYH* and *OGG1* mutations are involved in early-onset CRC in Japanese patients. *Methods.* Thirty-four Japanese patients with early-onset CRC were examined for germline *MUTYH* and *OGG1* mutations using sequencing. *Results.* Biallelic pathogenic mutations were not found in any of the patients; however, a heterozygous p.Arg19∗  *MUTYH* variant and a heterozygous p.Arg109Trp *MUTYH* variant were detected in one patient each. The p.Arg19∗ and p.Arg109Trp corresponded to p.Arg5∗ and p.Arg81Trp, respectively, in the type 2 nuclear-form protein. The defective DNA repair activity of p.Arg5∗ is apparent, while that of p.Arg81Trp has been demonstrated using DNA cleavage and *supF* forward mutation assays. *Conclusion.* These results suggest that biallelic *MUTYH* or *OGG1* pathogenic mutations are rare in Japanese patients with early-onset CRC; however, the p.Arg19∗ and p.Arg109Trp *MUTYH* variants are associated with functional impairments.

## 1. Introduction

8-Hydroxyguanine (8OHG) is an oxidized form of guanine, and the formation of 8OHG in DNA causes a G:C to T:A mutation, since 8OHG can pair with adenine as well as cytosine [1, 2]. To prevent such mutations in human cells, MUTYH (MIM #604933) and OGG1 (MIM #601982) proteins are involved in DNA glycosylase-initiating base excision repair, which is a component of the human DNA repair system [3–8]. MUTYH catalyzes the removal of adenine mispaired with 8OHG in double-stranded DNA, and OGG1 catalyzes the removal of 8OHG at the 8OHG:C site. Regarding MUTYH, multiple forms, including two major forms (type 1 mitochondrial form and type 2 nuclear form), are expressed in human cells [3–6].

Clinically, biallelic germline inactivating* MUTYH* mutations are known to predispose an individual to MUTYH-associated polyposis (MAP: MIM #608456), a hereditary disorder characterized by multiple colorectal polyps and carcinoma(s) [9–11]. Various pathogenic* MUTYH* mutations, including two major mutations (p.Tyr179Cys and p.Gly396Asp), have been detected in MAP patients in several ethnic populations [12]; however, neither the p.Tyr179Cys nor the p.Gly396Asp mutation has been found in the Japanese population [13–15], and which* MUTYH* variations are the major pathogenic mutations in the Japanese population remains unclear. In accordance with the notion that early-onset cancer is likely to be associated with the germline abnormality of certain gene(s) [16], early-onset colorectal cancer (CRC) is one of the characteristics of MAP [12], and early-onset CRC is thought to occur in patients with biallelic-inactivating mutations of another 8OHG repair gene,* OGG1*. Therefore, we hypothesized that biallelic inactivating mutations of* MUTYH* or* OGG1* might lead to early-onset CRC in Japanese patients. To test this hypothesis, we examined 34 Japanese patients with early-onset CRC for germline* MUTYH* and* OGG1 *mutations. Then, we investigated whether the detected variants were associated with defective 8OHG repair activity.

## 2. Materials and Methods

### 2.1. Clinical Samples and Cell Line

Blood specimens from 685 CRC cases and 778 controls were collected in a previous study [17]. Written informed consent was obtained from each individual patient [17]. The characteristics of the cases and the controls have been described previously [17, 18]. Briefly, the cases were composed of a consecutive series of patients with histologically confirmed incident colorectal adenocarcinomas, and the controls were composed of individuals that had no diagnosis of CRC. Additionally, the cases had no prior history of removal of the colorectum, familial adenomatous polyposis (FAP: MIM #175100), or inflammatory bowel disease (IBD). All the cases and the controls had been previously genotyped for c.36+11C>T, c.504+35A>G, c.934−2A>G, and c.1014G>C* MUTYH* polymorphisms [19], but none had been sequenced for the whole coding exons of the* MUTYH* and* OGG1* genes. A human cancer cell line, H1299, was obtained from the American Type Culture Collection (Manassas, VA). The H1299 cells and their derivatives were maintained at 37°C in RPMI1640 medium supplemented with 10% fetal bovine serum (Equitech-Bio, Kerrville, TX) and penicillin/streptomycin under a 5% CO_2_ atmosphere. The study design was approved by the institutional review boards.

### 2.2. PCR-Sequencing Analysis

Genomic DNA was extracted as described previously [17]. All the coding exons of the* MUTYH* and* OGG1* genes and their boundary regions were amplified using PCR with HotStarTaq DNA polymerase (Qiagen, Valencia, CA). The PCR primer sequences of* MUTYH* and* OGG1 *are summarized in Supplementary Tables S1 and S2 in the Supplementary Material available online at http://dx.doi.org/10.1155/2014/617351, respectively. The PCR-amplified products were directly sequenced with a BigDye Terminator Cycle Sequencing Reaction Kit (Applied Biosystems, Tokyo, Japan) and an ABI 3130 Genetic Analyzer (Applied Biosystems).

### 2.3. PCR with Confronting Two-Pair Primers (PCR-CTPP) Analysis

Genotyping of the c.55C>T and c.325C>T variants was performed using a PCR-CTPP analysis, as described previously [20]. The PCR primer sequences are summarized in Supplementary Table S3. The PCR products were fractionated by electrophoresis on a 2.0% agarose gel and were stained with ethidium bromide.

### 2.4. Construction of Expression Plasmid

Plasmid vectors for the expression of human wild-type (WT) MUTYH type 2 in* Escherichia coli *(*E. coli*) and human cells were previously constructed by inserting the cDNA into a pET25b(+) vector (Novagen, Darmstadt, Germany) and a piggyBac cumate switch inducible vector (System Biosciences, Mountain View, CA), respectively [21, 22]. Expression vectors for the MUTYH variants were generated using site-directed mutagenesis with a QuikChange Site-directed Mutagenesis kit (Stratagene, La Jolla, CA).

### 2.5. Preparation of Recombinant Protein

*E. coli* BL21-CodonPlus (DE3)-RP competent cells (Stratagene) were transformed with the MUTYH-pET25b vector and cultured at 37°C until an A_600_ of 0.6. After incubation with 0.1 mM IPTG at 15°C for 12 h, MUTYH-His_6_ protein was purified with TALON metal affinity resins (Clontech, Palo Alto, CA) and a TALON 2 mL disposable gravity column (Clontech). The protein was then dialyzed against buffer containing 10 mM sodium phosphate (pH 7.6), 50 mM NaCl, 0.5 mM DTT, 0.1 mM EDTA, 0.5 mM PMSF, 2 *μ*g/mL pepstatin, 2 *μ*g/mL leupeptin, 50 *μ*M chymostatin, and 10% glycerol. The quality and concentration of MUTYH proteins were determined by resolving the proteins with SDS-polyacrylamide gel electrophoresis (PAGE) and staining them with Coomassie Brilliant Blue (CBB); Image J software (National Institutes of Health, Bethesda, MD) was then used for quantification.

### 2.6. Western Blot Analysis

A Western blot analysis using a mouse anti-MUTYH monoclonal antibody (4D10; Abnova, Taipei, Taiwan) or an anti-*β*-tubulin monoclonal antibody (clone 2-28-33; Sigma-Aldrich, St. Louis, MO) was performed as described previously [22, 23].

### 2.7. DNA Cleavage Assay

First, 30-mer oligonucleotides containing or not containing a single 8OHG (5′-CTG GTG GCC TGA C[8OHG or T]C ATT CCC CAA CTA GTG-3′) were chemically synthesized and purified using PAGE (Japan Bio Services, Saitama, Japan). Complementary oligonucleotides containing an adenine opposite the 8OHG or T were ^32^P-labeled at the 5′ terminus with a MEGALABEL kit (Takara, Osaka, Japan) and [*γ*-^32^P]ATP (PerkinElmer, Tokyo, Japan), and these oligonucleotides were then annealed to oligonucleotides containing a single 8OHG or T. A reaction mixture containing 20 mM sodium phosphate (pH 7.6), 100 mM NaCl, 0.5 mM DTT, 0.5 mM EDTA, 5 *μ*M ZnCl_2_, 1.5% glycerol, 2.5 nM labeled oligonucleotide, 50 *μ*g/mL BSA, and 90 fmoles of MUTYH protein was then incubated at 37°C for 15 min, and the mixture was treated with 0.1 M NaOH. After the mixture was denatured, it was subjected to 20% PAGE. A ^32^P-labeled marker oligonucleotide was used as a size marker for the cleavage products. The radioactivities of the intact and cleaved oligonucleotides were quantified using an FLA-3000 fluoroimage analyzer (Fuji Film, Tokyo, Japan) and ImageGauge software (Fuji Film).

### 2.8. Establishment of Stable Inducible Cell Lines

H1299 cells were transfected with the piggyBac cumate switch inducible vector for MUTYH expression together with the piggyBac transposase vector (System Biosciences). To establish stable inducible cell lines, positively transposed cells were selected using puromycin (1 *μ*g/mL). Since the inducible piggyBac vector features a tight cumate switch combined with an EF1-CymR repressor-T2A-Puro cassette to establish stable cell lines, the addition of cumate solution (System Biosciences) to the puromycin-selected cells leads to the induction of MUTYH expression.

### 2.9. Indirect Immunofluorescence Analysis

Cells were fixed with 10% formalin at room temperature, permeabilized with 1% Nonidet P-40, blocked with 10% normal goat serum, and probed with mouse anti-MUTYH monoclonal antibody (4D10; Abnova). Indirect immunofluorescence labeling was performed by exposure to an Alexa Fluor 594-conjugated secondary antibody (Molecular Probes, Eugene, OR), and the nuclei were stained with 4′,6-diamidino-2-phenylindole (DAPI) (Sigma-Aldrich). The slides were promptly examined under a fluorescence microscope (Olympus BX-51-FL; Olympus, Tokyo, Japan) equipped with epifluorescence filters and a photometric CCD camera (Sensicam; PCO Company, Kelheim, Germany). The images captured were digitized and stored in the image analysis program (MetaMorph; Molecular Devices, Palo Alto, CA).

### 2.10. *supF* Forward Mutation Assay

A single 8OHG:C mispair was introduced at nucleotide position 159 of the bacterial suppressor tRNA (*supF*) gene of the shuttle vector plasmid pMY189, as described previously [22–24]. A* supF* forward mutation assay for H1299-derived cells was performed using the 8OHG-containing pMY189 plasmid and the KS40/pKY241 indicator* E. coli* strain, as described previously [22, 23]. The mutation frequencies were calculated as the number of* E. coli supF* mutants per total number of* E. coli* transformants. The mutations in the* supF* gene were then analyzed as described previously [22].

### 2.11. Computational Analysis for Variants

The functional effects of nonsynonymous variants were predicted by the online software tools PolyPhen-2 [25], SIFT [26], and PROVEAN [27]. The allele frequencies of variants in a large number of Japanese individuals were examined using a reference database of genetic variations in the Japanese population (http://www.genome.med.kyoto-u.ac.jp/SnpDB/).

### 2.12. Statistical Analysis

The statistical analyses were performed using an unpaired *t*-test, Dunnett test, or Fisher exact test. JMP version 9 software (SAS Institute, Cary, NC) was used for all the statistical analyses. *P* values less than 0.05 were considered statistically significant.

## 3. Results

### 3.1. Identification of p.Arg19* and p.Arg109Trp* MUTYH* Variants in Japanese Patients with Early-Onset CRC

**Figure 1:**
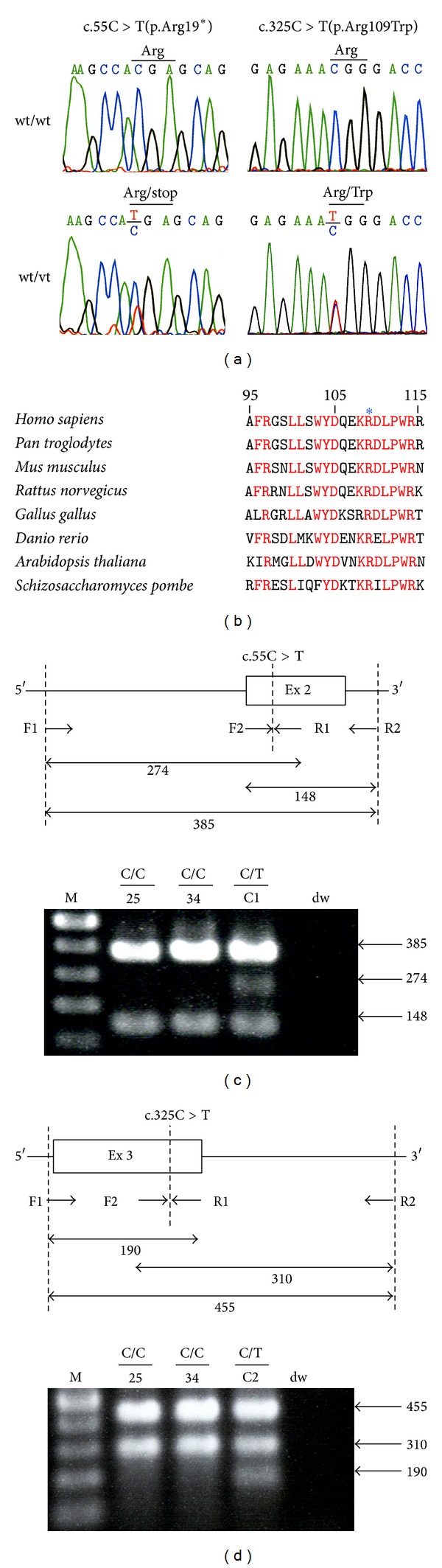
Identification and genotyping of c.55C>T (p.Arg19*) and c.325C>T (p.Arg109Trp) variants of the* MUTYH* gene in the Japanese population. (a) Identification of c.55C>T and c.325C>T variants of the* MUTYH* gene in Japanese patients with early-onset CRC. Sequencing electropherograms show a C to T variation at the c.55 and c.325 positions (lower panels). (b) Amino acid sequence alignment of a section of MUTYH among different species. The human MUTYH protein sequence from p.Ala95 to p.Arg115 was compared with the MUTYH sequences of other species. Amino acids exhibiting ≥75% identity among the species are shown in red. The position of p.Arg109 is marked by an asterisk. (c and d) Genotyping of the c.55C>T (c) and c.325C>T (d) variants of the* MUTYH *gene in Japanese individuals without CRC (control individuals). The schematic diagrams of PCR-CTPP used to genotype the c.55C>T and c.325C>T variants are shown in the upper panel. The PCR primers are indicated by the horizontal arrows; and F and R mean forward primer and reverse primer, respectively. The location of each variant is indicated by a vertical dashed line. The PCR product sizes for the primer pairs of F1 and R1, F2 and R2, and F1 and R2 are shown. Representative results of agarose gel electrophoresis of the PCR-CTPP products are shown in the lower panel. The number on the panel indicates the assigned number of control individuals, “C1” and “C2” indicate a case with a variant allele, and “dw” indicates the no template DNA in the PCR. M indicates a size marker.

To investigate whether germline mutations of the DNA glycosylase genes* MUTYH* and* OGG1* are involved in early-onset CRC in Japanese patients, we attempted to utilize a population of 685 Japanese CRC patients. Among them, we selected 34 CRC patients with the lowest ages of onset (corresponding to 5% of the total patient population) (Table 1). All 34 CRC patients were less than 43 years old and had no history of FAP or IBD. We then examined the 34 CRC patients for germline* MUTYH *and* OGG1* mutations by sequencing every coding exon of both genes. As a result, 9* MUTYH* variants and 7* OGG1* variants were found (Table 2, Figure 1(a)), and the genotype and allele frequencies of the nucleotide variations are summarized in Table 2 and the characteristics of the variations are summarized in Table 3. Genotypes of the variations in the coding region and splice-site region in 34 patients are also summarized in Supplementary Tables S4 and S5. Among the 9* MUTYH* variants, although c.36+11C>T, c.504+35A>G, c.934−2A>G, c.1014G>C (p.Gln338His), c.1118C>T (p.Ala373Val), c.1431G>C (p.Thr477Thr), and c.1477−40C>G are not considered to be MAP pathogenic alleles, the remaining two variants of c.55C >T (p.Arg19*) and c.325C>T (p.Arg109Trp) were identical to variants previously found in one patient each with colorectal multiple adenomas and a carcinoma [28]. In our study, the p.Arg19* variant was detected in one patient as a heterozygous status for the wild-type and variant alleles, and the p.Arg109Trp variant was also detected heterozygously in one patient. Moreover, the p.Arg19* variant encodes a truncating protein, and the p.Arg109Trp variant encodes a protein with the substitution of a highly conserved amino acid, p.Arg109 (National Center for Biotechnology Information, Bethesda, MD) (Figure 1(b)); the missense protein was predicted to be functionally damaged by the PolyPhen-2, SIFT, and PROVEAN software programs (Table 3). Therefore, we considered the two variants to be candidate MAP pathogenic alleles. Regarding the 7* OGG1* variants, c.949−89G>T and c.966C>T (p.Asp322Asp) were novel (Supplementary Figure S1); however, since the former exists in an intron and the latter is a synonymous variation, these variants were not considered to be pathogenic mutations. Among the other 5* OGG1* variants, c.−18G>T and c.977C>G (p.Ser326Cys) have been reported as low-penetrance cancer susceptibility variants [29, 30], while the remaining three were untranslated, synonymous, or intronic variations, meaning that all of them are not highly pathogenic mutations. Therefore, none of the patients were thought to have biallelic pathogenic* MUTYH* or* OGG1* mutations in the presently studied early-onset CRC group. However, we decided to further investigate the p.Arg19* and p.Arg109Trp* MUTYH* variants as candidate pathogenic alleles.

### 3.2. Absence of p.Arg19* and p.Arg109Trp* MUTYH* Variants in Japanese Individuals without CRC

To determine the frequency of the p.Arg19* and p.Arg109Trp* MUTYH* variants in Japanese individuals without CRC, we genotyped both nucleotide variations using PCR-CTPP in as many as 100 individuals randomly selected from the control group (Figures 1(c) and 1(d)). Since the above-described cases with a p.Arg19* or p.Arg109Trp variant exhibited a heterozygous pattern of the WT and variant alleles, these cases were used as positive controls in the PCR-CTPP analysis. The results showed that none of the controls exhibited p.Arg19* or p.Arg109Trp variants, suggesting that p.Arg19* and p.Arg109Trp are rare variants in the Japanese population.

### 3.3. Defective DNA Glycosylase Activity of MUTYH Type 2 p.Arg81Trp Variant

**Figure 2:**
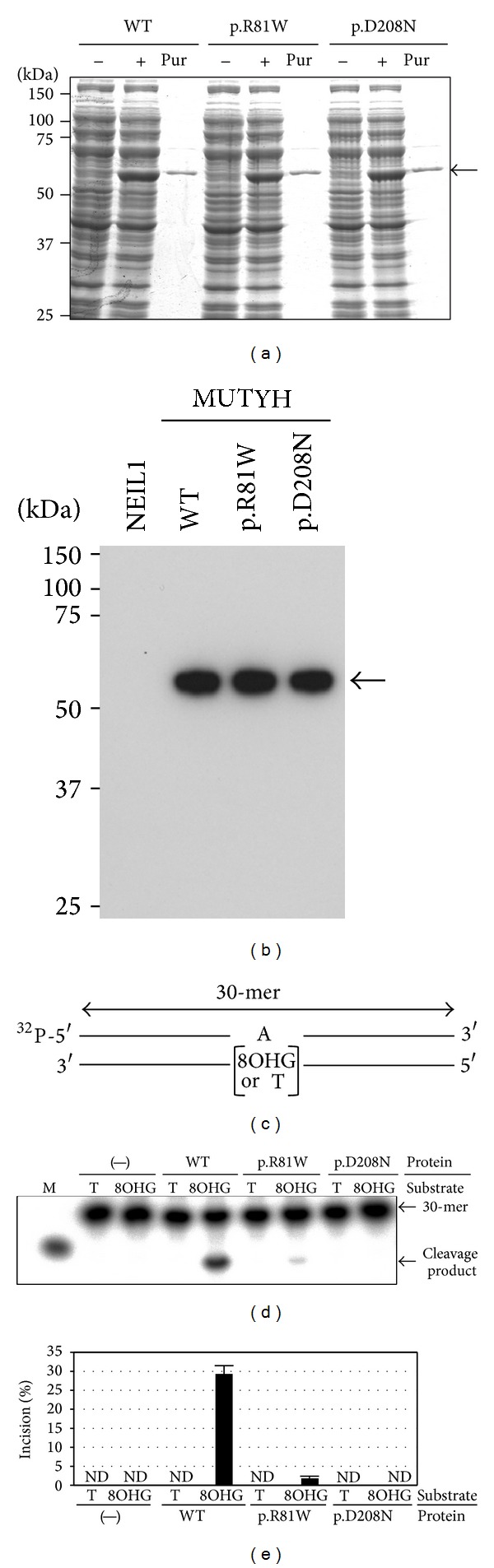
Comparison of the DNA glycosylase activity of WT MUTYH and p.Arg81Trp variant MUTYH protein using a DNA cleavage assay. (a) Purification of WT, p.Arg81Trp, and p.Asp208Asn MUTYH type 2 recombinant proteins. The MUTYH proteins were overexpressed and purified using the pET system and TALON metal affinity resins. Representative results for the expression and purification of MUTYH proteins resolved by SDS-PAGE and stained with CBB are shown. “−” and “+” mean the absence and presence, respectively, of IPTG induction, and “Pur” means purified MUTYH type 2 proteins. The arrow points to the MUTYH-His_6_ protein band. (b) Western blot of purified MUTYH type 2 proteins. MUTYH-His_6_ proteins are indicated by the arrow. Purified recombinant DNA glycosylase NEIL1-His_6_ protein, which was previously prepared using the same system as that used for the MUTYH-His_6_ protein [38], was included as a negative control. (c) Substrate used in the DNA cleavage assay. ^32^P-labeled 30-mer double-stranded oligonucleotides containing or not containing a single 8OHG mispair were prepared. (d) Measurement of the DNA glycosylase activity of WT, p.Arg81Trp, and p.Asp208Asn MUTYH type 2 protein on double-stranded DNA containing an 8OHG using the DNA cleavage assay. The reaction mixture was subjected to 20% PAGE. The intact 30-mer oligonucleotides and cleavage products are indicated by the arrows. “M” means a marker oligonucleotide. The amount of cleavage products as a proportion of the total oligonucleotides was calculated as the % incision, and the values are shown in (e). The values are the means ± standard errors of data from three independent experiments. ND means not detected.

To conclude whether a* MUTYH* variant allele is pathogenic, an impairment in the repair activity of the protein based on the nucleotide variant must be evident. p.Arg19* and p.Arg109Trp correspond to p.Arg5* and p.Arg81Trp, respectively, in the type 2 nuclear-form protein, which is the major MUTYH form in human cells. Since p.Arg5* has a length of only 5 amino acids, compared with 521 amino acids in the full-length type 2 protein, the DNA repair activity of p.Arg5* is most likely defective. On the other hand, p.Arg81Trp yields a missense form, and the repair activity of the mutant protein has not yet been analyzed. Thus, we planned to evaluate the DNA glycosylase activity of the p.Arg81Trp protein by comparing the cleavage activity of the mutant with that of the WT protein in the presence of an A:8OHG mismatch-containing DNA substrate. We also planned to utilize the p.Asp208Asn mutant as a negative control in the comparison [21]. First, the WT, p.Arg81Trp, and p.Asp208Asn recombinant proteins were expressed in* E. coli* and were purified to a high level of homogeneity (Figure 2(a)). Their molecular size of approximately 61 kDa was determined using SDS-PAGE/CBB staining and Western blot analysis, and this size corresponded to the size calculated from the cDNA sequence (Figures 2(a) and 2(b)). Then, the DNA glycosylase activity of the MUTYH proteins was examined by determining its capacity to cleave a double-stranded oligonucleotide containing an adenine mispaired with 8OHG (Figure 2(c)). No clear cleavage products were detected when an oligonucleotide containing an unmodified A:T base pair was exposed to any of the MUTYH proteins, but cleavage products with the same mobility as the marker oligonucleotide were detected when WT MUTYH protein, but not a p.Asp208Asn negative control protein, was allowed to react with an oligonucleotide containing an A:8OHG base pair (Figures 2(d) and 2(e)). Importantly, a significantly smaller amount of cleavage products was detected in the reaction with p.Arg81Trp than in the reaction with WT (% incision: 1.8% versus 29.3%) (Figures 2(d) and 2(e)). These results indicate that the DNA glycosylase activity of p.Arg81Trp was severely decreased.

### 3.4. Impaired Suppressive Activity of MUTYH Type 2 p.Arg81Trp Variant against Mutations Caused by 8OHG

**Figure 3:**
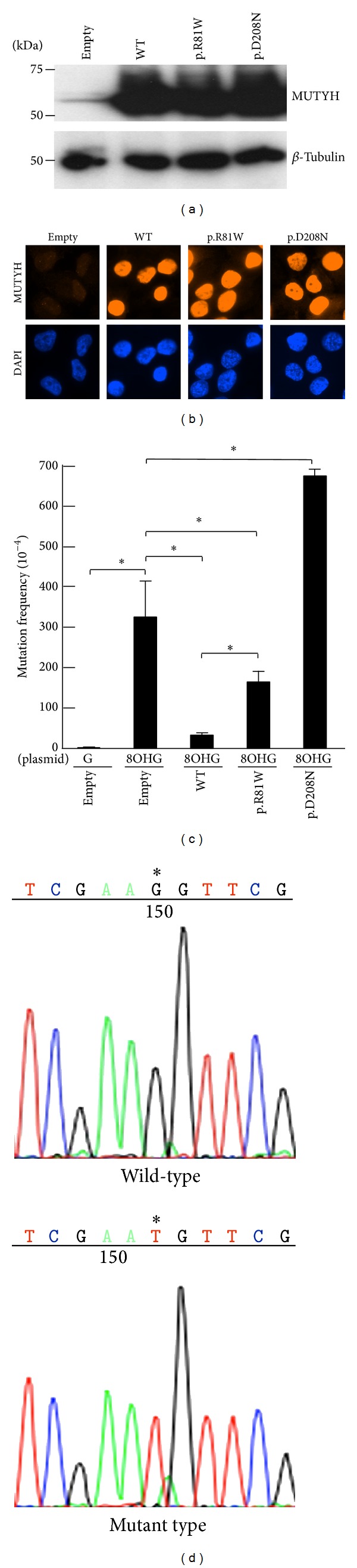
Comparison of the activity to suppress the mutation caused by 8OHG between H1299 human cell lines inducibly expressing WT MUTYH and p.Arg81Trp variant MUTYH protein using a* supF* forward mutation assay. (a) Detection of MUTYH proteins in cumate-inducible stable cell lines expressing MUTYH using a Western blot analysis with an anti-MUTYH antibody. Lysates from empty vector-transposed cells and cells inducibly expressing WT type 2 MUTYH, type 2 p.Arg81Trp MUTYH variant, or p.Asp208Asn negative control in the presence of cumate were analyzed. *β*-Tubulin protein was also analyzed as an internal control. (b) Immunofluorescence detection of MUTYH proteins expressed in the cell lines used in (a) in the presence of cumate. The MUTYH protein (red) was stained with anti-MUTYH as the primary antibody and Alexa Fluor 594-conjugated goat anti-mouse IgG as the secondary antibody. The nuclei were counterstained with DAPI (blue). (c) Measurement of the mutation frequency of the* supF* gene in the pMY189 plasmid using a* supF* forward mutation assay in H1299 human cell lines inducibly expressing MUTYH proteins. The cell lines used in (a) in the presence of cumate were transfected with a pMY189 shuttle plasmid, and the mutation frequency of* supF* in these human cell lines was measured. “8OHG” indicates a pMY189 plasmid containing an 8-hydroxyguanine residue at position 159 of* supF*, while “G” indicates a pMY189 plasmid containing the WT* supF* gene. The data are shown as the means ± standard error. (d) A representative result of a* supF* mutation in an 8OHG-containing pMY189 replicated in empty vector-transposed H1299 cells. Sequencing electropherograms show a G to T (G:C to T:A) mutation at position 159 (marked by asterisks) of the* supF *gene.

To investigate the ability of MUTYH p.Arg81Trp variant to suppress mutations caused by 8OHG in human cells, we planned to use the piggyBac transposon vector system [31] to establish human cells capable of inducibly expressing MUTYH protein and to perform a* supF* forward mutation assay using the shuttle plasmid pMY189, which contains a single 8OHG in the* supF* gene. First, we established human H1299 cell lines capable of inducibly expressing WT, p.Arg81Trp, or p.Asp208Asn MUTYH using the piggyBac transposon vector system [31]. The expression of MUTYH protein after cumate induction was examined using a Western blot analysis with an anti-MUTYH monoclonal antibody (Figure 3(a)). MUTYH protein was abundantly expressed in cells in which a WT, p.Arg81Trp, or p.Asp208Asn MUTYH expression vector, but not an empty vector, was transposed. An immunofluorescence analysis also showed abundant MUTYH protein expression in cells in which a WT, p.Arg81Trp, or p.Asp208Asn MUTYH expression vector, but not an empty vector, was transposed (Figure 3(b)). The p.Arg81Trp variant, as well as the WT MUTYH protein, was localized in the nucleus, suggesting that the amino acid changes in p.Arg81Trp were unlikely to alter the subcellular localization of the protein in human cells.

Next, the mutation frequencies were compared among the empty vector-transposed human cells and the cumate-inducible stable cells expressing WT or a variant MUTYH using a* supF* forward mutation assay with the shuttle plasmid pMY189. In this assay, we introduced a single 8OHG residue at position 159 of the* supF* gene in pMY189. The mutation frequency of* supF* was 3.2 × 10^−2^ in the 8OHG-containing pMY189 plasmid and 2.3 × 10^−4^ in the WT pMY189 in empty vector-transposed cells (Figure 3(c)), representing a 139-fold increase in the mutation frequency with the introduction of 8OHG. The mutation frequency of* supF* in the 8OHG-containing pMY189 plasmid in the WT MUTYH-transposed and p.Arg81Trp variant-transposed, but not p.Asp208Asn-transposed, cells was significantly lower than that in the empty vector-transposed cells (Figure 3(c)). Importantly, the* supF* mutation frequency in the p.Arg81Trp-transposed cells was significantly higher than that in the WT MUTYH-transposed cells (1.6 × 10^−2^ versus 3.3 × 10^−3^), meaning that the suppressive activity of the p.Arg109Trp variant against mutations caused by 8OHG in human cells was severely decreased when compared with that of WT MUTYH.

We further investigated what kind of mutation is contained in the* supF* mutant colony in the* supF* forward mutation assay. PCR and gel electrophoresis for the* supF* region of the mutants revealed that the percentage of mutant clones with the same mobility as a WT clone was significantly lower in the WT MUTYH-transposed cells (52%) than the empty vector-transposed cells and the p.Arg81Trp-transposed or p.Asp208Asn-transposed cells (>92%), meaning that the activity to decrease* supF* alterations of the base substitutions or small insertions/deletions caused by 8OHG is lower in the p.Arg81Trp variant than in WT MUTYH (Table 4). Further sequencing analysis of the mutants revealed that a G:C to T:A transversion at position 159 of* supF* among the 8OHG-containing pMY189 was predominant (>91%) in empty vector-transposed cells and p.Arg81Trp-transposed or p.Asp208Asn-transposed cells, while the proportion of the G:C to T:A transversion was significantly reduced in the WT MUTYH-transposed cells (46%) (Table 4, Figure 3(d), Supplementary Figure S2). These results suggest that the suppressive activity of the p.Arg81Trp variant against G:C to T:A mutations caused by 8OHG in human cells was severely reduced, compared with that of WT MUTYH.

## 4. Discussion

In this study, no biallelic pathogenic mutations were found in 34 Japanese patients with early-onset CRC, although a total of 9* MUTYH* variants and 7* OGG1* variants were detected. Among them, the p.Arg19* and p.Arg109Trp* MUTYH* variants were identical to variants previously reported by Vogt et al. [28] in non-Japanese patients with multiple colorectal adenomas and a carcinoma. According to Vogt's report, both the p.Arg19* and p.Ala385Profsx25 mutations were detected in one male patient with multiple (60–70) colorectal adenomas and a carcinoma, and both the p.Arg109Trp and p.Gly396Asp mutations were detected in another male patient with multiple (50–100) colorectal adenomas and a carcinoma. Since both patients had the clinical symptoms of MAP and p.Ala385Profsx25 and p.Gly396Asp are pathogenic mutations frequently found in non-Asian MAP patients [12, 28], p.Arg19* and p.Arg109Trp were speculated to be pathogenic mutations. The p.Arg19* and p.Arg109Trp variants correspond to p.Arg5* and p.Arg81Trp, respectively, in the type 2 form. p.Arg5* is strongly suspected to have a defective DNA repair activity because of its extremely short structure, but the p.Arg81Trp missense variant has not been functionally characterized. Therefore, we investigated type 2 p.Arg81Trp MUTYH using a DNA cleavage assay and a* supF* forward mutation assay and found that the abilities of p.Arg81Trp to cleave A:8OHG-containing DNA and to suppress mutations caused by 8OHG were severely reduced. These results suggest that biallelic* MUTYH* or* OGG1* pathogenic mutations are very rare or nonexistent in Japanese patients with early-onset CRC; however, they also suggest that the* MUTYH* alleles of p.Arg19* and p.Arg109Trp detected in our patient series are associated with functional impairment. This information would be of great help in diagnosing MAP worldwide, judging from the existence of alleles in both Japanese and other ethnicities.

p.Tyr179Cys and p.Gly396Asp are major pathogenic* MUTYH* mutations for MAP in many ethnicities other than Asian, and some ethnic-specific* MUTYH* mutations, for example, p.Glu480del (Southern Europe), p.Tyr104* (Pakistan), and p.Glu480* (India), have been reported [12, 32]. Regarding pathogenic* MUTYH* mutations in the Japanese population, p.Gly286Glu is the only* MUTYH* mutation for which the resulting protein was experimentally shown to be defective in DNA repair activity and to be found in the Japanese population [14]. The p.Gly286Glu mutation was found as a homozygous mutation in a Japanese patient with colorectal multiple polyps and a carcinoma by Yanaru-Fujisawa et al. [14], and in the paper, mouse MUTYH mutant protein corresponding to the human p.Gly286Glu was shown to have an impaired repair activity. However, this mutation has not been detected in other* MUTYH* mutation screenings performed in Japanese CRC patients [13–15], including the current study, and whether the p.Gly286Glu pathogenic mutation is common in the Japanese population remains unclear. The p.Arg19* detected in our analysis was previously found as a heterozygous mutation in one Japanese patient with CRC reported by Kuno et al. [15], suggesting that it could be relatively common impaired* MUTYH* mutation in the Japanese population. On the other hand, the p.Arg109Trp also detected in our analysis is the first demonstration of such a variant in the Japanese population. Since neither the p.Arg19* nor the p.Arg109Trp variation was observed in our screening of 100 Japanese control individuals, these variants are considered to be relatively rare among the general Japanese population. In addition to the fact that the two major pathogenic* MUTYH* mutations of p.Tyr179Cys and p.Gly396Asp have not been seen in Japanese individuals in previous studies [13–15] or the present study, the p.Arg19* and p.Arg109Trp variations as well as the p.Gly286Glu variation, rather than the p.Tyr179Cys and p.Gly396Asp variations, are thought to account for functionally impaired* MUTYH* alleles in the Japanese population. A combination of these* MUTYH* variations would cause an even higher susceptibility to MAP.

The type 2 MUTYH protein is a nuclear form of MUTYH [4–6], and somatic* AP*C (MIM #611731) and* KRA*S (MIM #190070) mutations occur in the nuclear DNA of MAP tumors [9, 10, 33]; therefore, we believed that it would be more appropriate to use type 2, rather than type 1, in a comparative study of MUTYH variants, and we analyzed the DNA repair function of the variant type 2 form* in vitro* and* in vivo* in this study. As a result, an impaired cleavage activity of type 2 p.Arg81Trp towards A:8OHG-containing DNA was clearly demonstrated using a DNA cleavage assay, and a severely reduced activity of the protein to suppress mutations caused by 8OHG in human cells was also clearly revealed using a* supF* forward mutation assay. A combination of the results of two distinct analyses, that is,* in vitro *and* in vivo *analyses, would provide more definitive proof of the pathogenicity of the p.Arg109Trp (type 2 p.Arg81Trp) MUTYH variant. The existence of a patient with multiple colorectal adenomas and a carcinoma, who carried both the p.Arg109Trp variant and the p.Gly396Asp pathogenic mutation, in the report by Vogt et al. [28] also supports the pathogenicity of the p.Arg109Trp MUTYH variant. Because the diagnosis of MAP depends on whether (1) the clinical phenotypic characteristics of MAP are present in a candidate patient; and (2) the repair activities of the MUTYH variant proteins encoded by the two* MUTYH *alleles of the patient are severely reduced, when* MUTYH *gene variations are found in the patient by mutation screening, information on the levels of the repair activities of the MUTYH variant proteins is indispensable for the proper diagnosis of MAP. Thus, our evaluation of the repair activity of the p.Arg109Trp (type 2 p.Arg81Trp) MUTYH variant is clinically useful.

So far, no analyses of the crystal structure of the full-length human MUTYH polypeptide have been reported; therefore, it is difficult to explain fully why an amino acid substitution in p.Arg109Trp leads to a functional impairment. However, p.Arg109 in human MUTYH protein is conserved among* Homo sapiens, Pan troglodytes*,* Mus musculus*,* Rattus norvegicus*,* Gallus gallus, Danio rerio, Arabidopsis thaliana, *and* Schizosaccharomyces pombe* (Figure 1(b)). Furthermore, mutations resulting in an amino acid exchange from Arg to Trp in codon 185 or 241 have been previously revealed to be pathogenic mutations by functional analyses [34, 35]. In addition, the PolyPhen-2, SIFT, and PROVEAN programs predicted that an amino acid substitution in p.Arg109Trp would alter its protein function (Table 3). Moreover, the screening for nonacceptable polymorphisms (SNAP) program, which predicts the effect of single amino acid substitutions on protein function (http://www.rostlab.org/services/SNAP) [36], also predicted that the MUTYH type 2 p.Arg81Trp variation was nonneutral. In conjunction with the fact that other various single missense* MUTYH* mutations also exist as pathogenic mutations [12, 33, 37], the notion that p.Arg109Trp is a functionally impaired allele is thought to be acceptable. In the future, a crystal structure analysis of the full-length MUTYH protein and its covalent complex with DNA, in conjunction with the present findings regarding the p.Arg109Trp variant, should contribute to establishing further correlations between the structure and repair function of the MUTYH protein.

The p.Arg19* MUTYH variant was detected heterozygously in a patient diagnosed with CRC at 43 years of age and a pathological stage of IIIa, while the p.Arg109Trp* MUTYH* variant was detected heterozygously in a patient diagnosed with CRC at 43 years of age and a pathological stage of I, as summarized in Table 1. The histological classification of CRCs of both patients was well-differentiated adenocarcinoma. Regarding their colorectal polyp status, both patients were recorded as non-FAP, and no other information was available. Therefore, we concluded that the two patients were unlikely to have exhibited any specific clinicopathological characteristics other than early-onset CRC.

In this paper, no biallelic pathogenic mutations in the* MUTYH* and* OGG1* genes were found in 34 Japanese patients with early-onset CRC. Since the sample size was relatively small, we could not make a robust conclusion; however, this result suggests that biallelic* MUTYH* or* OGG1* pathogenic mutations are very rare or possibly nonexistent in Japanese patients with early-onset CRC. A future study with a large number of Japanese patients with early-onset CRC is needed to obtain a robust conclusion regarding this issue.

In conclusion, our results suggested that biallelic* MUTYH* or* OGG1* pathogenic mutations are rare among Japanese patients with early-onset CRC; however, they also suggested that the p.Arg19* and p.Arg109Trp* MUTYH* variants that were detected in our Japanese patient group are functionally impaired alleles. This information is likely to be very useful in the diagnosis of MAP worldwide. Additionally, since recent technological progress in genome sequencing analysis has contributed to efficient and rapid genome screening, an increase in the number of novel* MUTYH* variants can be expected in the future. Our analysis system for determining the repair abilities of MUTYH variants, as successfully performed in this study, might be useful for characterizing such newly detected variants.

## Supplementary Material

We provided the following Supplementary Tables S1-S5: Supplementary Table S1 (Primers used for PCR amplification of the *MUTYH* gene), Supplementary Table S2 (Primers used for PCR amplification of the *OGG1* gene), Supplementary Table S3 (Primers used for PCR-CTPP of the *MUTYH* c.55C>T and c.325C>T variants), Supplementary Table S4 (*MUTYH* nucleotide variations in its coding region and splice-site region found in 34 Japanese patients with early-onset CRC), and Supplementary Table S5 (*OGG1* nucleotide variations in its coding region found in 34 Japanese patients with early-onset CRC).
We also provided the following Supplementary Figures S1 and S2: Supplementary Figure S1 *[*Identification of novel *OGG1* variants of c.949-89G>T and c.966C>T (p.Asp322Asp) in Japanese patients with early-onset CRC by sequencing analysis*]* and Supplementary Figure S2 *[*The distribution of *supF* base substitution-type mutations in a *supF* forward mutation assay using the pMY189 plasmid containing 8-hydroxyguanine (8OHG) at position 159 of *supF* in H1299 human cell lines inducibly expressing *MUTYH* protein*]*.

## Figures and Tables

**Table 1:** Clinicopathological profiles of 34 Japanese patients with early-onset CRC.

Case number	Age	Sex	Tumor site	Tumor histology	Tumor stage
1	27	F	Distal colon	Mucinous adenocarcinoma	II
2	29	F	Proximal colon	WD adenocarcinoma^a^	II
3	34	F	Distal colon	WD adenocarcinoma	I
4	36	M	Distal colon	WD adenocarcinoma	IIIa
5	39	F	Rectum	MD adenocarcinoma^b^	IIIa
6	39	F	Distal colon	WD adenocarcinoma	IV
7	38	M	Proximal colon	MD adenocarcinoma	II
8	36	F	Distal colon	MD adenocarcinoma	IIIb
9	39	F	Rectum	WD adenocarcinoma	IIIa
10	39	M	Proximal colon	WD adenocarcinoma	IIIb
11	37	F	Proximal colon	WD adenocarcinoma	I
12	33	F	Proximal colon	WD adenocarcinoma	IIIa
13	36	M	Rectum	WD adenocarcinoma	I
14	35	M	Distal colon	MD adenocarcinoma	II
15	37	M	Proximal colon	MD adenocarcinoma	IIIb
16	38	M	Rectum	WD adenocarcinoma	II
17	43	M	Rectum	WD adenocarcinoma	I
18	41	F	Distal colon	WD adenocarcinoma	II
19	41	M	Distal colon	MD adenocarcinoma	IIIb
20	42	M	Rectum	WD adenocarcinoma	IIIb
21	40	M	Proximal colon	WD adenocarcinoma	I
22	43	F	Proximal colon	MD adenocarcinoma	II
23	42	M	Rectum	MD adenocarcinoma	IIIa
24	42	M	Proximal colon	MD adenocarcinoma	I
25	42	M	Rectum	WD adenocarcinoma	I
26	43	M	Rectum	PD adenocarcinoma^c^	IV
27	43	M	Distal colon	WD adenocarcinoma	IIIa
28	41	M	Rectum	MD adenocarcinoma	II
29	42	M	Rectum	WD adenocarcinoma	IIIa
30	40	M	Rectum	MD adenocarcinoma	II
31	43	M	Distal colon	WD adenocarcinoma	I
32	43	M	Distal colon	WD adenocarcinoma	II
33	42	M	Proximal colon	WD adenocarcinoma	II
34	40	M	Rectum	MD adenocarcinoma	IIIb

^a^Well-differentiated adenocarcinoma. ^b^Moderately differentiated adenocarcinoma. ^c^Poorly differentiated adenocarcinoma.

**Table 2:** Genotype and allele frequencies of the *MUTYH* and *OGG1* nucleotide variations found in 34 Japanese patients with early-onset colorectal carcinoma.

Gene	Variant	Nucleotide		Genotype frequency			Allele frequency	
		wt	vt	wt/wt	wt/vt	vt/vt	wt	vt
*MUTYH *	c.36+11C>T	C	T	32 (94.1%)	2 (5.9%)	0 (0%)	66 (97.1%)	2 (2.9%)
*MUTYH *	c.55C>T (p.Arg19*)	C	T	33 (97.1%)	1 (2.9%)	0 (0%)	67 (98.5%)	1 (1.5%)
*MUTYH *	c.325C>T (p.Arg109Trp)	C	T	33 (97.1%)	1 (2.9%)	0 (0%)	67 (98.5%)	1 (1.5%)
*MUTYH *	c.504+35A>G	A	G	25 (73.5%)	9 (26.5%)	0 (0%)	59 (86.8%)	9 (13.2%)
*MUTYH *	c.934−2A>G	A	G	33 (97.1%)	1 (2.9%)	0 (0%)	67 (98.5%)	1 (1.5%)
*MUTYH *	c.1014G>C (p.Gln338His)	G	C	15 (44.1%)	15 (44.1%)	4 (11.8%)	45 (66.2%)	23 (33.8%)
*MUTYH *	c.1118C>T (p.Ala373Val)	C	T	33 (97.1%)	1 (2.9%)	0 (0%)	67 (98.5%)	1 (1.5%)
*MUTYH *	c.1431G>C (p.Thr477Thr)	G	C	32 (94.1%)	2 (5.9%)	0 (0%)	66 (97.1%)	2 (2.9%)
*MUTYH *	c.1477−40C>G	C	G	2 (5.9%)	7 (20.6%)	25 (73.5%)	11 (16.2%)	57 (83.8%)
*OGG1 *	c.−23A>G	A	G	32 (94.1%)	2 (5.9%)	0 (0%)	66 (97.1%)	2 (2.9%)
*OGG1 *	c.−18G>T	G	T	32 (94.1%)	2 (5.9%)	0 (0%)	66 (97.1%)	2 (2.9%)
*OGG1 *	c.294G>A (p.Lys98Lys)	G	A	33 (97.1%)	1 (2.9%)	0 (0%)	67 (98.5%)	1 (1.5%)
*OGG1 *	c.748−15C>G	C	G	13 (38.2%)	14 (41.2%)	7 (20.6%)	40 (58.8%)	28 (41.2%)
*OGG1 *	c.949−89G>T	G	T	33 (97.1%)	1 (2.9%)	0 (0%)	67 (98.5%)	1 (1.5%)
*OGG1 *	c.966C>T (p.Asp322Asp)	C	T	33 (97.1%)	1 (2.9%)	0 (0%)	67 (98.5%)	1 (1.5%)
*OGG1 *	c.977C>G (p.Ser326Cys)	C	G	11 (32.4%)	17 (50.0%)	6 (17.6%)	39 (57.4%)	29 (42.6%)

wt: wild-type, vt: variant type.

**Table 3:** Characteristics of the *MUTYH* and *OGG1* nucleotide variations found in 34 Japanese patients with early-onset colorectal carcinoma.

Gene	Variant	Position^a^	dbSNP ID^b^	PolyPhen-2 prediction (score)^d^	SIFT prediction (score)^d^	PROVEAN prediction (score)^d^	Allele frequency in a Japanese SNP database^e^
*MUTYH *	c.36+11C>T	45805880	rs2275602	—	—	—	0.048
*MUTYH *	c.55C>T (p.Arg19*)	45800165	NA^c^	—	—	—	0.002
*MUTYH *	c.325C>T (p.Arg109Trp)	45799108	NA	Probably damaging (1)	Damaging (0)	Deleterious (−7.22)	NS^f^
*MUTYH *	c.504+35A>G	45798555	rs3219487	—	—	—	0.12
*MUTYH *	c.934−2A>G	45797760	rs77542170	—	—	—	0.026
*MUTYH *	c.1014G>C (p.Gln338His)	45797505	rs3219489	Benign (0.343)	Tolerated (0.136)	Neutral (−1.03)	0.434
*MUTYH *	c.1118C>T (p.Ala373Val)	45797401	rs35352891	Possibly damaging (0.506)	Tolerated (0.128)	Neutral (−2.324)	0.01
*MUTYH *	c.1431G>C (p.Thr477Thr)	45796899	rs74318065	—	—	—	0.051
*MUTYH *	c.1477−40C>G	45796269	rs3219493	—	—	—	0.885
*OGG1 *	c.−23A>G	9791948	rs1801129	—	—	—	0.039
*OGG1 *	c.−18G>T	9791953	rs1801126	—	—	—	0.033
*OGG1 *	c.294G>A (p.Lys98Lys)	9792785	rs1801127	—	—	—	0.015
*OGG1 *	c.748−15C>G	9798140	rs2072668	—	—	—	0.452
*OGG1 *	c.949−89G>T	9798656	NA	—	—	—	NS
*OGG1 *	c.966C>T (p.Asp322Asp)	9798762	NA	—	—	—	NS
*OGG1 *	c.977C>G (p.Ser326Cys)	9798773	rs1052133	Benign (0.121)	Tolerated (0.176)	Neutral (−0.647)	0.446

^a^Genome positions of *MUTYH* and *OGG1* variants are shown according to the reference sequences (GRCh37) of chromosome 1 and chromosome 3, respectively. ^b^Identification number of variants according to the database of single nucleotide polymorphisms (dbSNP) located on the homepage of the National Center for Biotechnology Information web site (http://www.ncbi.nlm.nih.gov/SNP/). ^c^NA, not assigned. ^d^The accession numbers for the reference proteins of MUTYH and OGG1 are E5KP25 and O15527, respectively. ^e^Variant allele frequency in a reference database of genetic variations in the Japanese population (http://www.genome.med.kyoto-u.ac.jp/SnpDB/). ^f^NS, not shown.

**Table 4:** *supF* mutations in a *supF* forward mutation assay using the pMY189 plasmid containing 8-hydroxyguanine (8OHG) at position 159 of *supF *in H1299 human cell lines inducibly expressing MUTYH protein*. *

Cell line^a^	Plasmid^b^	PCR and gel electrophoresis		Sequencing				
		Number of mutant clones analyzed	Number of mutant clones showing the same mobility as a WT clone (%)	Number of mutant clones analyzed	Number of mutant clones containing a mutation at position 159 of *supF* (%)			
					Total	G:C to T:A	G:C to A:T	G:C to C:G
Empty	G (WT)	22	3 (13.6)	3	0 (0)	0 (0)	0 (0)	0 (0)
Empty	8OHG	40	38 (95.0)^c^	24	24 (100)	22 (91.7)^d^	1 (4.2)	1 (4.2)
WT	8OHG	25	13 (52.0)^c^	13	8 (61.5)	6 (46.2)^d^	1 (7.7)	1 (7.7)
p.R81W	8OHG	40	37 (92.5)^c^	24	23 (95.8)	22 (91.7)^d^	0 (0)	1 (4.2)
p.D208N	8OHG	40	38 (95.0)^c^	24	23 (95.8)	23 (95.8)^d^	0 (0)	0 (0)

^a^Empty vector-transposed H1299 human cancer cell line and H1299 cells inducibly expressing type 2 MUTYH protein of WT, p.Arg81Trp, or p.Asp208Asn were used.

^b^The shuttle plasmid pMY189, containing 8-hydroxyguanine (8OHG) at nucleotide position 159 of *supF*, or a wild-type (WT) pMY189 plasmid was used.

^c^The *P* value for the difference in the proportion between cells transfected with an 8OHG-containing pMY189 plasmid was <0.0001 (Fisher exact test).

^d^The *P* value for the difference in the proportion between cells transfected with an 8OHG-containing pMY189 plasmid was <0.001 (Fisher exact test).

## References

[1] Kasai H, Nishimura S (1984). Hydroxylation of deoxyguanosine at the C-8 position by ascorbic acid and other reducing agents. *Nucleic Acids Research*.

[2] Shibutani S, Takeshita M, Grollman AP (1991). Insertion of specific bases during DNA synthesis past the oxidation-damaged base 8-oxodG. *Nature*.

[3] Slupska MM, Luther WM, Chiang J-H, Yang H, Miller JH (1999). Functional expression of hMYH, a human homolog of the *Escherichia coli* MutY protein. *Journal of Bacteriology*.

[4] Takao M, Zhang Q-M, Yonei S, Yasui A (1999). Differential subcellular localization of human MutY homolog (hMYH) and the functional activity of adenine:8-oxoguanine DNA glycosylase. *Nucleic Acids Research*.

[5] Shinmura K, Yamaguchi S, Saitoh T (2000). Adenine excisional repair function of MYH protein on the adenine:8-hydroxyguanine base pair in double-stranded DNA. *Nucleic Acids Research*.

[6] Ohtsubo T, Nishioka K, Imaiso Y (2000). Identification of human MutY homolog (hMYH) as a repair enzyme for 2-hydroxyadenine in DNA and detection of multiple forms of hMYH located in nuclei and mitochondria. *Nucleic Acids Research*.

[7] David SS, O’Shea VL, Kundu S (2007). Base-excision repair of oxidative DNA damage. *Nature*.

[8] Cai Z, Chen H, Tao J (2012). Association of base excision repair gene polymorphisms with ESRD risk in a Chinese population. *Oxidative Medicine and Cellular Longevity*.

[9] Al-Tassan N, Chmiel NH, Maynard J (2002). Inherited variants of *MYH* associated with somatic G : C→T : A mutations in colorectal tumors. *Nature Genetics*.

[10] Jones S, Emmerson P, Maynard J (2002). Biallelic germline mutations in *MYH * predispose to multiple colorectal adenoma and somatic G : C→T : A mutations. *Human Molecular Genetics*.

[11] Sieber OM, Lipton L, Crabtree M (2003). Multiple colorectal adenomas, classic adenomatous polyposis, and germ-line mutations in *MYH*. *New England Journal of Medicine*.

[12] Nielsen M, Morreau H, Vasen HF, Hes FJ (2011). *MUTYH*-associated polyposis (MAP). *Critical Reviews in Oncology/Hematology*.

[13] Miyaki M, Iijima T, Yamaguchi T (2005). Germline mutations of the *MYH* gene in Japanese patients with multiple colorectal adenomas. *Mutation Research—Fundamental and Molecular Mechanisms of Mutagenesis*.

[14] Yanaru-Fujisawa R, Matsumoto T, Ushijima Y (2008). Genomic and functional analyses of *MUTYH* in Japanese patients with adenomatous polyposis. *Clinical Genetics*.

[15] Kuno T, Matsubara N, Tsuda S (2012). Alterations of the base excision repair gene *MUTYH* in sporadic colorectal cancer. *Oncology Reports*.

[16] Bleyer A, Barr R, Hayes-Lattin B (2008). The distinctive biology of cancer in adolescents and young adults. *Nature Reviews Cancer*.

[17] Kono S, Toyomura K, Yin G, Nagano J, Mizoue T (2004). A case-control study of colorectal cancer in relation to lifestyle factors and genetic polymorphisms: design and conduct of the fukuoka colorectal cancer study. *Asian Pacific Journal of Cancer Prevention*.

[18] Hagiwara T, Kono S, Yin G (2005). Genetic polymorphism in cytochrome P450 7A1 and risk of colorectal cancer: the Fukuoka colorectal cancer study. *Cancer Research*.

[19] Tao H, Shinmura K, Suzuki M (2008). Association between genetic polymorphisms of the base excision repair gene *MUTYH* and increased colorectal cancer risk in a Japanese population. *Cancer Science*.

[20] Tao H, Shinmura K, Hanaoka T (2004). A novel splice-site variant of the base excision repair gene *MYH* is associated with production of an aberrant mRNA transcript encoding a truncated *MYH* protein not localized in the nucleus. *Carcinogenesis*.

[21] Goto M, Shinmura K, Nakabeppu Y (2010). Adenine DNA glycosylase activity of 14 Human MutY homolog (*MUTYH*) variant proteins found in patients with colorectal polyposis and cancer. *Human Mutation*.

[22] Shinmura K, Goto M, Tao H, Matsuura S, Matsuda T, Sugimura H (2012). Impaired suppressive activities of human *MUTYH* variant proteins against oxidative mutagenesis. *World Journal of Gastroenterology*.

[23] Shinmura K, Goto M, Suzuki M (2011). Reduced expression of *MUTYH* with suppressive activity against mutations caused by 8-hydroxyguanine is a novel predictor of a poor prognosis in human gastric cancer. *Journal of Pathology*.

[24] Matsuda T, Yagi T, Kawanishi M, Matsui S, Takebe H (1995). Molecular analysis of mutations induced by 2-chloroacetaldehyde, the ultimate carcinogenic form of vinyl chloride, in human cells using shuttle vectors. *Carcinogenesis*.

[25] Adzhubei IA, Schmidt S, Peshkin L (2010). A method and server for predicting damaging missense mutations. *Nature Methods*.

[26] Kumar P, Henikoff S, Ng PC (2009). Predicting the effects of coding non-synonymous variants on protein function using the SIFT algorithm. *Nature Protocols*.

[27] Choi Y, Sims GE, Murphy S, Miller JR, Chan AP (2012). Predicting the functional effect of amino acid substitutions and indels. *PLoS ONE*.

[28] Vogt S, Jones N, Christian D (2009). Expanded extracolonic tumor spectrum in *MUTYH*-associated polyposis. *Gastroenterology*.

[29] Ishida T, Takashima R, Fukayama M (1999). New DNA polymorphisms of human *MMH/OGG1* gene: prevalence of one polymorphism among lung-adenocarcinoma patients in Japanese. *International Journal of Cancer*.

[30] Xu Z, Yu L, Zhang X (2013). Association between the hOGG1 Ser326Cys polymorphism and lung cancer susceptibility: a meta-analysis based on 22, 475 subjects. *Diagnostic Pathology*.

[31] Ding S, Wu X, Li G, Han M, Zhuang Y, Xu T (2005). Efficient transposition of the piggyBac (PB) transposon in mammalian cells and mice. *Cell*.

[32] Aretz S, Genuardi M, Hes FJ (2013). Clinical utility gene card for: *MUTYH*-associated polyposis (MAP), autosomal recessive colorectal adenomatous polyposis, multiple colorectal adenomas, multiple adenomatous polyps (MAP)—update 2012. *European Journal of Human Genetics*.

[33] Lipton L, Halford SE, Johnson V (2003). Carcinogenesis in *MYH*-associated polyposis follows a distinct genetic pathway. *Cancer Research*.

[34] Bai H, Jones S, Guan X (2005). Functional characterization of two human MutY homolog (hMYH) missense mutations (R227W and V232F) that lie within the putative hMSH6 binding domain and are associated with hMYH polyposis. *Nucleic Acids Research*.

[35] D’Agostino VG, Minoprio A, Torreri P (2010). Functional analysis of *MUTYH* mutated proteins associated with familial adenomatous polyposis. *DNA Repair*.

[36] Bromberg Y, Rost B (2007). SNAP: predict effect of non-synonymous polymorphisms on function. *Nucleic Acids Research*.

[37] Shinmura K, Goto M, Tao H, Sugimura H (2012). Role of base excision repair enzyme *MUTYH* in the repair of 8-hydroxyguanine and *MUTYH*-associated polyposis (MAP). *Hereditary Genetics*.

[38] Shinmura K, Tao H, Goto M (2004). Inactivating mutations of the human base excision repair gene *NEIL1* in gastric cancer. *Carcinogenesis*.

