# Supplementary material for: Impaired 8-Hydroxyguanine Repair Activity of MUTYH Variant p.Arg109Trp Found in a Japanese Patient with Early-Onset Colorectal Cancer
Source: Oxid Med Cell Longev. 2014 Mar 23;2014:617351. doi: 10.1155/2014/617351 (PMC3988950; doi:10.1155/2014/617351)

Supplementary Information

for

Impaired 8-hydroxyguanine repair activity of MUTYH variant p.Arg109Trp found in a Japanese patient with early-onset colorectal cancer

Supplementary Tables S1-S5

Supplementary Table S1:

Primers used for PCR amplification of the *MUTYH* gene

Supplementary Table S2:

Primers used for PCR amplification of the *OGG1* gene

Supplementary Table S3:

Primers used for PCR-CTPP of the *MUTYH* c.55C>T and c.325C>T variants

Supplementary Table S4:

MUTYH nucleotide variations in its coding region and splice-site region found in 34 Japanese patients with early-onset CRC

Supplementary Table S5:

OGG1 nucleotide variations in its coding region found in 34 Japanese patients with early-onset CRC

Supplementary Figures S1 and S2

Supplementary Figure S1:

Identification of novel *OGG1* variants of c.949-89G>T and c.966C>T (p.Asp322Asp) in Japanese patients with early-onset CRC by sequencing analysis

Supplementary Figure S2:

The distribution of *supF* base substitution-type mutations in a *supF* forward mutation assay using the pMY189 plasmid containing 8-hydroxyguanine (8OHG) at position 159 of *supF* in H1299 human cell lines inducibly expressing MUTYH protein

Supplementary Table S1: Primers used for PCR amplification of the *MUTYH* gene.

Amplicon number	Forward sequence (5'->3')	Reverse sequence (5'->3')	Product size (bp)
1	gaagctgcgggagctgaaac	cggcgacccgacggcgagac	160
2	ttggcctcattgtgactgac	gcacctggcccttagtaagtc	280
3	aggctgctgtgtcccaagac	gaggacactgctgacctgcc	444
4	ggcaggtcagcagtgtcctc	ccctagctcctctaccacctg	542
5	ggtaggaacccaggagtcttg	agagctcctttgcagacacc	406
6	ctgcaaaggagctctgcttc	ctttaggggccaacctagagag	463
7	taggttggcccctaaagccc	gcccaacgctgtagttcctg	448
8	tggcaggactgtgggagttc	caacagagcgattctccgtc	488
9	tgggattataggcgtgagcc	acctatggactcaggcctgg	239
10	tcccccaactacaaggcctc	aacatagcgagacccccatctc	278

Supplementary Table S2: Primers used for PCR amplification of the *OGG1* gene.

Amplicon number	Forward sequence (5'->3')	Reverse sequence (5'->3')	Product size (bp)
1	cttaagggtcgtggtccttg	gaggggacaggcttctcag	268
2	catgttgctcagactggaagatag	cttatgtccaagaaccctaaccc	427
3	ctctaacggtgctgactctcatt	gaacagatcttgaaagctgatgg	367
4	cttgaagatgcctgatgcttg	ctttctccttcctgctctgc	346
5	gggctataagcaagatgctgg	gagaagtttctaccatcccagc	274
6	cacctctccctcagaccctac	tggggaatttctttgtccag	493
7	cttgtgcaggacagcaatctc	atttcattcccaagcaggc	473

Supplementary Table S3: Primers used for PCR-CTPP of the *MUTYH* c.55C>T and c.325C>T variants.

Variant	Primer name[a]	Primer sequence (5'->3')[b]
c.55C>T	F1	CTTTGCATGTCTCCAGGGCTG
c.55C>T	F2	GGCCATCATGAGGAAGCCA**C**
c.55C>T	R1	CACTTCCCACGGCTGCTC**A**
c.55C>T	R2	CCTTGGGCCACAACCTAGTTCC
c.325C>T	F1	AGCCTGTGCAGGGATGATT
c.325C>T	F2	CTAAGCTGGTACGACCAAGAGAAA**C**
c.325C>T	R1	TCTTCTCCATGGTAGGTCCC**A**
c.325C>T	R2	CATGACCTCTGAGACCCACAC

[a]This primer name is corresponded to that in Figure 1C,D.

[b]Nucleotide at the variant position is shown in bold.

Supplementary TABLE S4: *MUTYH* nucleotide variations in its coding region and splice-site region found in 34 Japanese patients with early-onset CRC

Case No.	c.55C>T (p.Arg19*)	c.325C>T (p.Arg109Trp)	c.934-2A>G	c.1014G>C (p.Gln338His)	c.1118C>T (p.Ala373Val)	c.1431G>C (p.Thr477Thr)
1	wt/wt	wt/wt	wt/wt	wt/wt	wt/wt	wt/wt
2	wt/wt	wt/wt	wt/wt	wt/wt	wt/wt	wt/wt
3	wt/wt	wt/wt	wt/wt	wt/wt	wt/wt	wt/wt
4	wt/wt	wt/wt	wt/wt	wt/vt	wt/wt	wt/wt
5	wt/wt	wt/wt	wt/wt	wt/vt	wt/wt	wt/wt
6	wt/wt	wt/wt	wt/wt	vt/vt	wt/wt	wt/wt
7	wt/wt	wt/wt	wt/vt	wt/vt	wt/wt	wt/wt
8	wt/wt	wt/wt	wt/wt	wt/vt	wt/wt	wt/wt
9	wt/wt	wt/wt	wt/wt	wt/wt	wt/wt	wt/wt
10	wt/wt	wt/wt	wt/wt	wt/vt	wt/wt	wt/vt
11	wt/wt	wt/wt	wt/wt	wt/vt	wt/wt	wt/vt
12	wt/wt	wt/wt	wt/wt	wt/vt	wt/wt	wt/wt
13	wt/wt	wt/wt	wt/wt	vt/vt	wt/wt	wt/wt
14	wt/wt	wt/wt	wt/wt	wt/vt	wt/wt	wt/wt
15	wt/wt	wt/wt	wt/wt	wt/wt	wt/wt	wt/wt
16	wt/wt	wt/wt	wt/wt	wt/wt	wt/wt	wt/wt
17	wt/wt	wt/wt	wt/wt	wt/vt	wt/wt	wt/wt
18	wt/wt	wt/wt	wt/wt	wt/vt	wt/wt	wt/wt
19	wt/wt	wt/wt	wt/wt	wt/wt	wt/wt	wt/wt
20	wt/wt	wt/wt	wt/wt	wt/wt	wt/wt	wt/wt
21	wt/wt	wt/wt	wt/wt	wt/wt	wt/wt	wt/wt
22	wt/wt	wt/wt	wt/wt	wt/wt	wt/wt	wt/wt
23	wt/wt	wt/wt	wt/wt	wt/vt	wt/wt	wt/wt
24	wt/wt	wt/wt	wt/wt	wt/wt	wt/vt	wt/wt
25	wt/wt	wt/wt	wt/wt	wt/wt	wt/wt	wt/wt
26	wt/wt	wt/wt	wt/wt	vt/vt	wt/wt	wt/wt
27	wt/vt	wt/wt	wt/wt	wt/vt	wt/wt	wt/wt
28	wt/wt	wt/wt	wt/wt	wt/wt	wt/wt	wt/wt
29	wt/wt	wt/wt	wt/wt	vt/vt	wt/wt	wt/wt
30	wt/wt	wt/wt	wt/wt	wt/wt	wt/wt	wt/wt
31	wt/wt	wt/vt	wt/wt	wt/vt	wt/wt	wt/wt
32	wt/wt	wt/wt	wt/wt	wt/vt	wt/wt	wt/wt
33	wt/wt	wt/wt	wt/wt	wt/vt	wt/wt	wt/wt
34	wt/wt	wt/wt	wt/wt	wt/wt	wt/wt	wt/wt

wt: wild type, vt: variant type.

Genotype containing variant type is shown in red font.

Supplementary TABLE S5: *OGG1* nucleotide variations in its coding region found in 34 Japanese patients with early-onset CRC.

Case No.	c.294G>A (p.Lys98Lys)	c.966C>T (p.Asp322Asp)	c.977C>G (p.Ser326Cys)
1	wt/wt	wt/wt	wt/vt
2	wt/wt	wt/wt	vt/vt
3	wt/wt	wt/wt	wt/wt
4	wt/wt	wt/wt	wt/vt
5	wt/wt	wt/wt	wt/vt
6	wt/wt	wt/wt	wt/vt
7	wt/wt	wt/wt	wt/vt
8	wt/wt	wt/wt	wt/wt
9	wt/wt	wt/wt	wt/wt
10	wt/wt	wt/wt	wt/wt
11	wt/vt	wt/wt	wt/wt
12	wt/wt	wt/wt	wt/wt
13	wt/wt	wt/wt	wt/vt
14	wt/wt	wt/wt	wt/vt
15	wt/wt	wt/wt	vt/vt
16	wt/wt	wt/wt	wt/vt
17	wt/wt	wt/wt	wt/wt
18	wt/wt	wt/wt	wt/wt
19	wt/wt	wt/wt	wt/vt
20	wt/wt	wt/wt	vt/vt
21	wt/wt	wt/wt	vt/vt
22	wt/wt	wt/wt	wt/vt
23	wt/wt	wt/wt	wt/wt
24	wt/wt	wt/wt	wt/vt
25	wt/wt	wt/wt	vt/vt
26	wt/wt	wt/wt	vt/vt
27	wt/wt	wt/wt	wt/vt
28	wt/wt	wt/vt	wt/vt
29	wt/wt	wt/wt	wt/wt
30	wt/wt	wt/wt	wt/vt
31	wt/wt	wt/wt	wt/vt
32	wt/wt	wt/wt	wt/wt
33	wt/wt	wt/wt	wt/vt
34	wt/wt	wt/wt	wt/vt

wt: wild type, vt: variant type.

Genotype containing variant type is shown in red font.

LEGENDS FOR SUPPLEMENTARY FIGURES

Supplementary Figure S1: Identification of novel *OGG1* variants of c.949-89G>T and c.966C>T (p.Asp322Asp) in Japanese patients with early-onset CRC by sequencing analysis. Sequencing electropherograms show a G>T and C>T nucleotide variation at c.949-89 and c.966 position, respectively (lower panels). wt: wild type, vt: variant type. These *OGG1* nucleotide variations have not been reported to date, meaning they are novel *OGG1* variants.

Supplementary Figure S2: The distribution of *supF* base substitution-type mutations in a *supF* forward mutation assay using the pMY189 plasmid containing 8-hydroxyguanine (8OHG) at position 159 of *supF* in H1299 human cell lines inducibly expressing MUTYH protein. Empty vector-transposed cells and cells inducibly expressing WT type 2 MUTYH, type 2 p.Arg81Trp MUTYH variant, or p.Asp208Asn negative control in the presence of cumate were analyzed.

Supplementary Figure S1

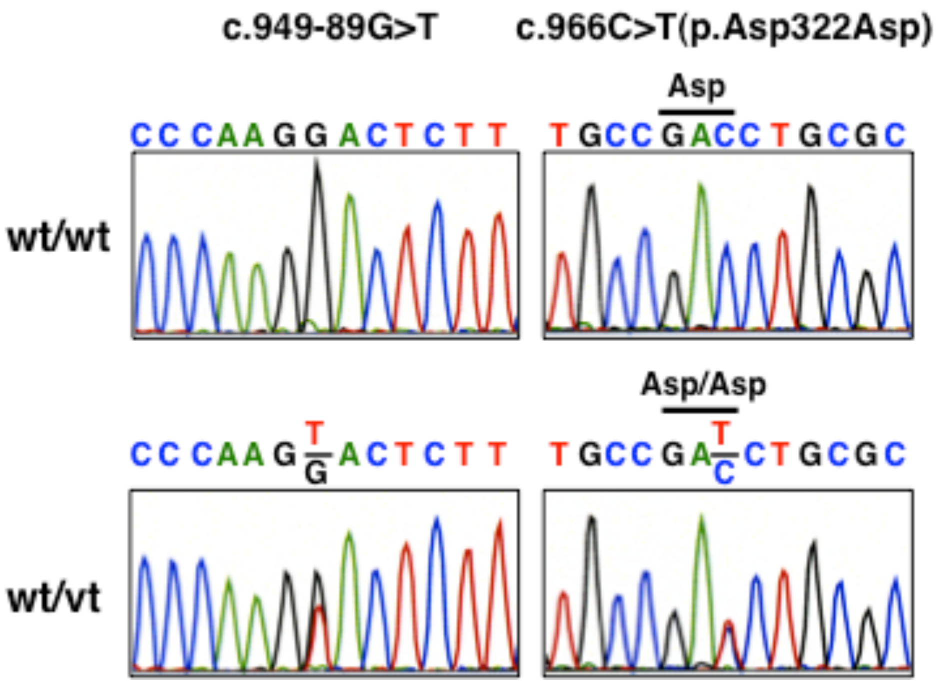

Supplementary Figure S2

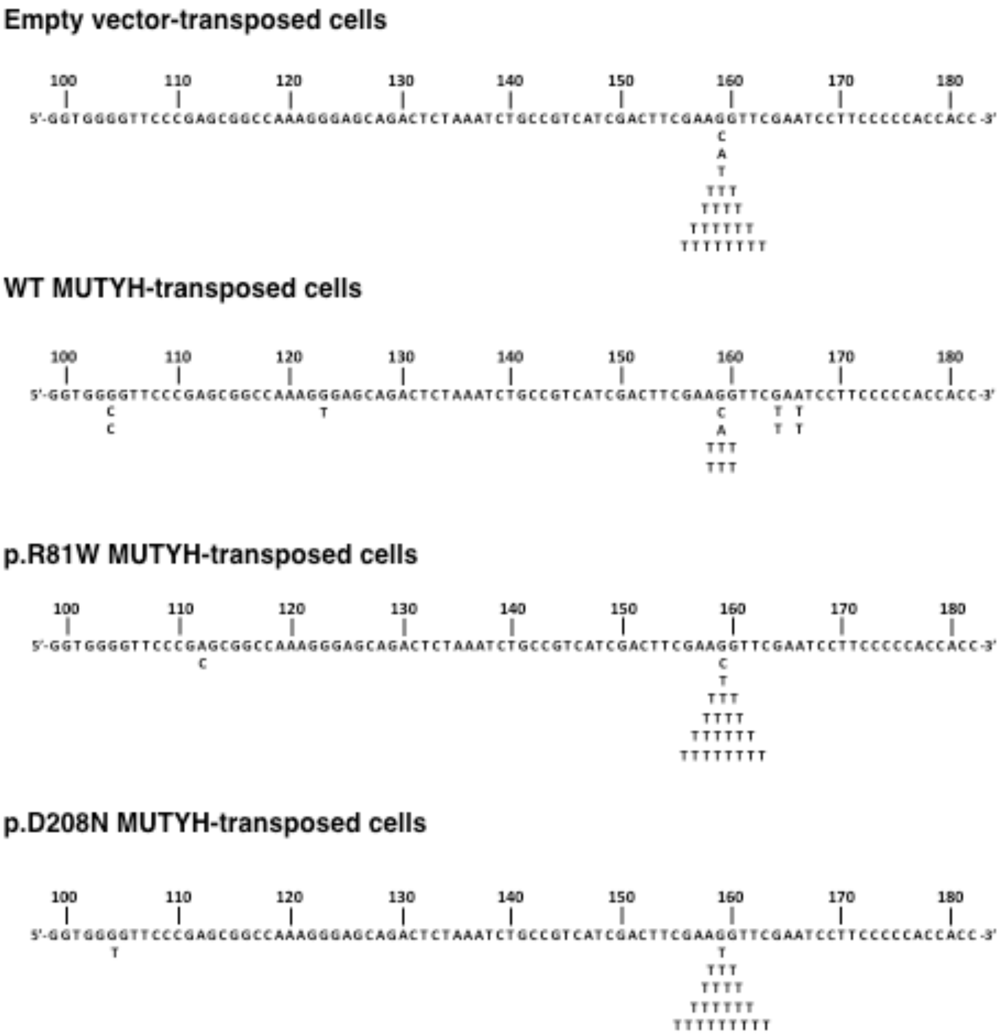

Supplement: Supplementary file 1 — We provided the following Supplementary Tables S1-S5: Supplementary Table S1 (Primers used for PCR amplification of the MUTYH gene), Supplementary Table S2 (Primers used for PCR amplification of the OGG1 gene), Supplementary Table S3 (Primers used for PCR-CTPP of the MUTYH c.55C>T and c.325C>T variants), Supplementary Table S4 (MUTYH nucleotide variations in its coding region and splice-site region found in 34 Japanese patients with early-onset CRC), and Supplementary Table S5 (OGG1 nucleotide variations in its coding region found in 34 Japanese patients with early-onset CRC). We also provided the following Supplementary Figures S1 and S2: Supplementary Figure S1 [Identification of novel OGG1 variants of c.949-89G>T and c.966C>T (p.Asp322Asp) in Japanese patients with early-onset CRC by sequencing analysis] and Supplementary Figure S2 [The distribution of supF base substitution-type mutations in a supF forward mutation assay using the pMY189 plasmid containing 8-hydroxyguanine (8OHG) at position 159 of supF in H1299 human cell lines inducibly expressing MUTYH protein]. [file 617351.f1.pdf]
